# Rush Medical College Commencement

**Published:** 1885-02

**Authors:** 


RUSH MEDICAL COLLEGE COMMENCEMENT.

The regular Annual Commencement exercises of the Rush Medical College, of this city, took place in Central Music Hall, on the afternoon of Feb. 17th, 1885.

The hall was well filled with an appreciative audience, and the ceremonies were of the usual formal and imposing character. The degrees were conferred by the President of the College, Professor J. Adams Allen, and the general valedictory address was delivered by Professor W. H. Byford. From the report of the Secretary of the Faculty, Professor J. H. Etheridge, we learn the whole number of students attending the past College term was 401, and the number on which degrees were conferred was 151; the length of the College term, a little less than five months.

